# Fluocinolone Acetonide Implant for Uveitis: Dissecting Responder and Non-Responder Outcomes at a Tertiary Center

**DOI:** 10.3390/biomedicines12051106

**Published:** 2024-05-16

**Authors:** Jasmin Abu Arif, Vitus André Knecht, Anne Rübsam, Vanessa Lussac, Zohreh Jami, Dominika Pohlmann, Bert Müller, Uwe Pleyer

**Affiliations:** 1 Department of Ophthalmology, Charité—Universitätsmedizin Berlin, Corporate Member of Freie Universität Berlin, Humboldt-Universität zu Berlin and Berlin Institute of Health, 10117 Berlin, Germany; jasminaarif04@gmail.com (J.A.A.); vitus-andre.knecht@charite.de (V.A.K.); anne.ruebsam@charite.de (A.R.); zohreh.jami@charite.de (Z.J.); dominika.pohlmann@charite.de (D.P.); bert.mueller@charite.de (B.M.); 2 Berlin Institute of Health at Charité—Universitätsmedizin Berlin, Charité Platz 1, 10117 Berlin, Germany

**Keywords:** dexamethasone, fluocinolone intravitreal implant, ILUVIEN, intraocular inflammation, macular edema, non-infectious uveitis, OZURDEX, pars plana vitrectomy (PPV), sustained release corticosteroid, visual acuity

## Abstract

Macular edema (ME) remains a primary cause of visual deterioration in uveitis. Visual acuity (VA) can often be maintained using corticosteroid depot systems. This study evaluated the efficacy of a fluocinolone acetonide (FAc) intravitreal implant (ILUVIEN^®^) in treating non-infectious uveitis using real-world data. This retrospective analysis included 135 eyes subdivided into responders and non-responders. Central retinal thickness (CRT), VA, and intraocular pressure (IOP) were followed over time. A significant decrease in CRT and an increase in VA were observed in all eyes throughout the follow-up period (*p* < 0.01). An IOP increase (*p* = 0.028) necessitated treatment in 43% of eyes by Month 6. Non-responders were older (*p* = 0.004) and had been treated with more dexamethasone (DEX) implants (*p* = 0.04); 89.3% had a defect in the external limiting membrane (ELM) and inner/outer segment (IS/OS) zone (*p* < 0.001). Immunomodulatory therapy had no impact on treatment response. Pars plana vitrectomy (PPV) patients had a mean CRT reduction of 47.55 µm and a reduced effect by Month 24 (*p* = 0.046) versus non-PPV patients. We conclude that the FAc implant achieves long-term control of CRT and improves VA. Increases in IOP were manageable. Eyes with a previous PPV showed milder results. Data showed a correlation between older age, a damaged ELM and IS/OS zone, frequent DEX inserts, and poorer outcome measures.

## 1. Introduction

Uveitis considerably impairs vision and remains one of the leading causes of blindness in the developed world [1]. Today, the primary approach to the management of non-infectious uveitis (NIU) comprises corticosteroids (CSs). Administration methods are adjusted according to the severity and nature of the disease and include topical, periocular, intraocular, and systemic approaches [2] As many studies have shown, CSs are associated with multiple side effects ranging from cataracts, glaucoma, and blindness to diabetes, Cushing’s syndrome, and hypercholesterolemia [2,3,4,5]. Systemic immunosuppressants represent a secondary line of treatment consisting of antimetabolites (methotrexate and azathioprine), calcineurinic inhibitors (cyclosporine and tacrolimus), and alkylating agents (cyclophosphamide and chlorambucil) [2,6]. Substantial numbers of patients do not tolerate or are refractory to this therapy, leading to the next option on the stepladder approach of therapy: biologic agents [7,8]. Adalimumab is the most frequently used biologic for treating NIU [9,10]. Nevertheless, real-world data, e.g., from the HOPE study, indicate that this therapy is ineffective in up to 30% of patients [11]. Non-responsiveness to the agent, the adverse effects of tumor necrosis factor alpha (TNF-α) inhibitors, and the development of anti-drug antibodies represent major limitations to this therapy [12,13]. 

In any prolonged treatment scenario, questions regarding safety, cost-effectiveness, and patient compliance naturally arise [14]. Sustained-release corticosteroid implants have revolutionized the treatment of NIU affecting the posterior segment of the eye. Indeed, the MUST study using fluocinolone acetonide highlighted significant improvements in visual acuity (VA) and the quality of life of patients undergoing this therapy [15]. The use of fluocinolone acetonide (FAc) intravitreal implants is licensed for the treatment of diabetic macular edema (DME) and the prevention of recurrent NIU affecting the posterior segment of the eye. Over a three-year period, it led to substantial visual benefits and an improvement in central retinal thickness (CRT) [16,17]. Some off-label studies suggest that FAc may also be beneficial in the treatment of macular edema (ME) associated with retinitis pigmentosa [18], pseudophakic cystoid macular edema (PCME) [19], and non-diabetic cystoid macular edema [20].

The most frequently used intravitreal implant is dexamethasone (DEX) (OZURDEX^®^), which is composed of a biodegradable polylactic acid–glycolic acid matrix. Due to its biodegradability, it completely dissolves, but its effects are limited to last for up to 6 months. In comparison, the FAc implant is composed of a drug–polymer matrix within a non-biodegradable cylindrical tube (measuring 3.5 mm × 0.37 mm), and its therapeutic effects are maintained for up to 36 months [21,22,23]. While both DEX and FAc avoid systemic immunosuppression and its associated side effects, the FAc implant has significant advantages due to the sustained release of fluocinolone acetonide [21]. Its long-term efficacy reduces the need for regular re-injections and does not allow for significant fluctuations in ME. This is considered important as fluctuating ME has been suggested to negatively impact VA [24]. Interestingly, the biological effects of DEX and FAc differ, with FAc showing a significantly higher glucocorticoid receptor affinity compared to DEX, which may relate to benefits such as enhanced efficacy, lower required dosages, and a sustained effect [25].

Conversely, since DEX is less lipophilic and therefore does not accumulate in the trabecular meshwork and lens as much as FAc, the incidence of adverse events, such as an increased risk for an intraocular pressure (IOP) increase and cataracts, could be lower [26,27,28,29]. However, lipophilicity is also dose-dependent, with higher doses being more likely to cause increased IOP [30]. The total dose of DEX is almost four times higher than that of FAc [30].

Although several reports that have indicated the benefit of FAc [31,32,33], important questions still remain unanswered. Indeed, past research focused on the effects of single FAc applications, meaning that the efficacy of re-injections with respect to the stability of CRT, functional outcomes, and long-term prognosis remains unclear. Also, little is known about morphological changes, such as ME, gliosis, and changes in the external limiting membrane (ELM) and inner and outer segment (IS/OS) zone, following FAc treatment and its impact on patients’ prognosis. Furthermore, a substantial number of patients may have undergone pars plana vitrectomy (PPV), therefore influencing the bioavailability of fluocinolone, something that we sought to address in this research. Previous reports showed that not all patients responded to Fac, and so we analyzed the characteristics of responders and non-responders and report them here. 

This paper will present the long-term outcomes of patients from a tertiary uveitis center located in Germany and will explore questions regarding the effectiveness of the FAc implant by assessing responders and non-responders to therapy, specifically (1) the long-term effects of FAc therapy on patient morphological and functional outcomes, (2) the effects of morphological parameters on patient outcome measures (ME, gliosis, and the ELM and IS/OS zone), (3) the efficacy of the FAc implant in patients who underwent PPV, and (4) the potential reasons for non-responsiveness to therapy. 

## 2. Materials and Methods

This retrospective study was conducted with the approval of the ethics commission of Charité—University Medicine Berlin, EA1/188/23. Medical data were collected from patient records, and imaging data were extracted from the optical coherence tomography (OCT) (Heidelberg Engineering GmbH 69115 Heidelberg, Germany) Heidelberg electronic medical database of Charité—University Medicine Berlin.

This study’s inclusion criteria consisted of patients diagnosed with non-infectious uveitis and PCME as well as uveitis associated with juvenile idiopathic arthritis (JIA) who received an FAc injection. We excluded patients who received the FAc implant for conditions other than non-infectious uveitis, such as diabetic retinopathy.

Descriptive patient data included age, sex, and number of previous DEX injections. The type of uveitis was also recorded for each patient. Data are available upon reasonable request.

Clinical data on VA, IOP, and the need for local IOP-lowering therapy were collected at specific intervals. This included the clinical visit before the implant (baseline) followed by control visits at Months 1, 3, 6, 12, 24, and 36 after administration, according to the follow-up data available. At times, the available data did not align with the preset schedule, mainly because of the COVID-19 pandemic.

For this reason, we had to introduce a two-month permissible time window for the collection of each data point until the twelfth month and a four-month permissible time window at the later time points (visits in Months 24 and 36). Objective evaluations were prioritized throughout data collection. 

Simultaneously, data were collected via OCT (SPECTRALIS^®^ OCT, Heidelberg Engineering GmbH) at predetermined time intervals. These imaging data included an assessment of CRT, the presence of ME, the presence of gliosis, and the integrity of the external limiting membrane (ELM) and the inner segment/outer segment (IS/OS) zone. The device’s software automatically determines the CRT in μm, measuring the distance from the inner limiting membrane (ILM) to the retinal pigment epithelium (RPE) at the most pronounced point, situated within a circle with a 1 mm radius around the fovea [34]. 

Firstly, we analyzed all eyes in our study to provide an overview of changes in the parameters over time. Based on the collected data, the eyes were categorized into two subgroups. Group 1, also referred to from now as the “responder” group, represented treated eyes with a successful long-term response to therapy with a minimum follow-up of 24 months. The responder group was represented by patients who benefited from the FAc therapy and demonstrated sustained positive outcomes throughout the follow-up period. No additional therapeutic interventions were required to control the NIU in this group. Group 2 was considered the “non-responder” group due to persistent or recurrent ME. It included eyes that required supportive therapy before the 24-month follow-up visit due to an insufficient response to FAc. These patients received additional DEX implants, the re-administration of FAc within 24 months of the initial implant, intravitreal triamcinolone, anti-vascular endothelial growth factor (anti-VEGF), or even retina surgery. Monitoring of these eyes continued even after these additional interventions. The modification of parameters over time in all groups was represented using graphs for a better overview of the changes.

### Statistical Analysis

We assessed data normality using the Shapiro–Wilk test and histograms. The paired-sample *t*-test was applied for normally distributed data, while the Wilcoxon signed-rank test was used for non-normal distributions to compare medians. A categorical data analysis involved the Chi-squared test and Fisher’s Exact Test. A Linear Regression Analysis was used to examine the impact of PPV on CRT. Longitudinal trends in anti-glaucoma medication use were analyzed using Generalized Estimating Equations (GEEs). No missing data substitutions were made; missing data were excluded pairwise in all tests. A *p*-value of ≤0.05 was considered significant. Analyses were conducted using Python 3.12, and figures were created using GraphPad Prism version 10.1.1. (GraphPad Software, La Jolla, CA, USA).

## 3. Results

### 3.1. Demographics and Clinical Characteristics

We included a total of one hundred thirty-five eyes from eighty-one patients. Seventeen eyes from eleven patients underwent a re-injection of FAc, of which one eye received three re-injections and one patient received one re-injection in both eyes. The patients were predominantly female (92 eyes/43 patients). The most common subtype of intraocular inflammation was intermediate uveitis (34.81%). The second most common subtype was posterior uveitis (34.07%). The majority of eyes (86%) were pseudophakic prior to FAc therapy. Around 28% of eyes had a previous PPV, and approximately 41% of eyes were linked to patients receiving systemic therapy (Adalimumab, Azathioprine, Certolizumab Pegol, Cyclosporine A, Golimumab, Infliximab, Methotrexate, Methylprednisolone, Mycophenolic acid, Prednisolone, Secukinumab, Tacrolimus, Tocilizumab, Tofacitinib, Upadacitinib, and Ustekinumab), as shown in Table 1.

The responder group (Group 1) had consistent, long-term follow-up, with N = 61 at the beginning, N = 57 after 24 months, and N = 27 after 36 months. Twenty-eight eyes were categorized as non-responders, of which sixteen eyes reached a follow-up of 24 months and twelve eyes reached a follow-up of 36 months. The patients in the non-responder group (Group 2) had a significantly higher average age in comparison to the responder group (*p* = 0.0046) and had received a significantly higher number of previous DEX intravitreal implants (*p* = 0.0487), as can be seen in Table 1. There was no significant difference regarding gender, systemic immunomodulatory therapy, and PPV between the groups. The difference between the two groups regarding lens status and the type of uveitis could not be assessed due to the small numbers of eyes in some of the categories.

### 3.2. Changes in Central Retinal Thickness

**Figure 1:**
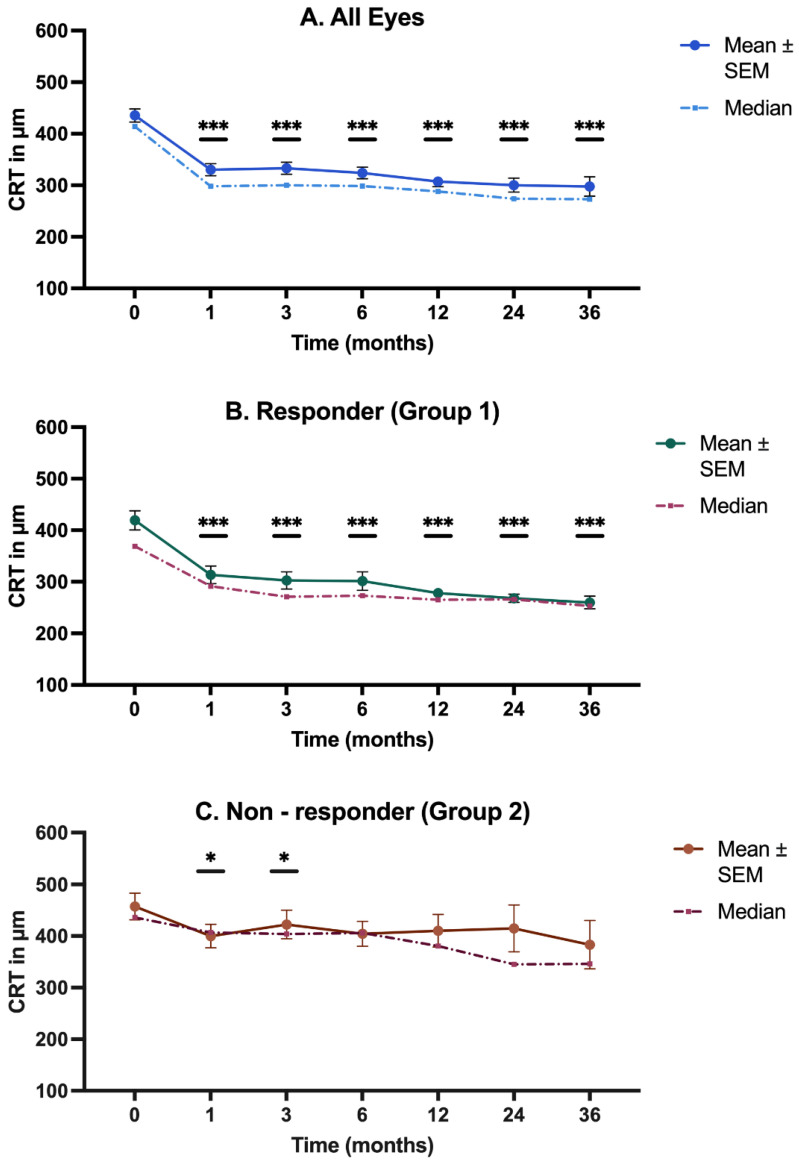
Temporal progression of central retinal thickness (CRT). The figure shows the progression of CRT measured at various time points over 36 months. (**A**) The temporal progression of CRT in all eyes; (**B**) The temporal progression of CRT in the responder group (Group 1); (**C**) The temporal progression of CRT in the non-responder group (Group 2). CRT = central retinal thickness; SEM = standard error of the mean. * *p* < 0.05; *** *p* < 0.001.

Figure 1A (representing all eyes) clearly illustrates a highly significant reduction in CRT throughout all follow-up months. The most substantial decrease was noticeable from baseline to Month 1 (*p* < 0.001), with the median CRT declining by 116 µm. In the subsequent follow-up months, the value continued to gradually decrease.

Group 1 (depicted in Figure 1B) indicated a strong decrease in CRT, with significant differences to baseline at all follow-up months (*p* < 0.001) and with an overall decrease being observed from a median of 369 µm at Month 0 to 253 µm at Month 36. 

Group 2 (Figure 1C) experienced a less pronounced CRT reduction than the responder group, demonstrating significance in only the first three months (*p* = 0.03). In the following months, the decrease was no longer statistically significant. The decrease in Month 1 was milder in the non-responder group (a median CRT reduction of 29 µm) than the responder group (a reduction of 78 µm).

### 3.3. Changes in Visual Acuity

**Figure 2:**
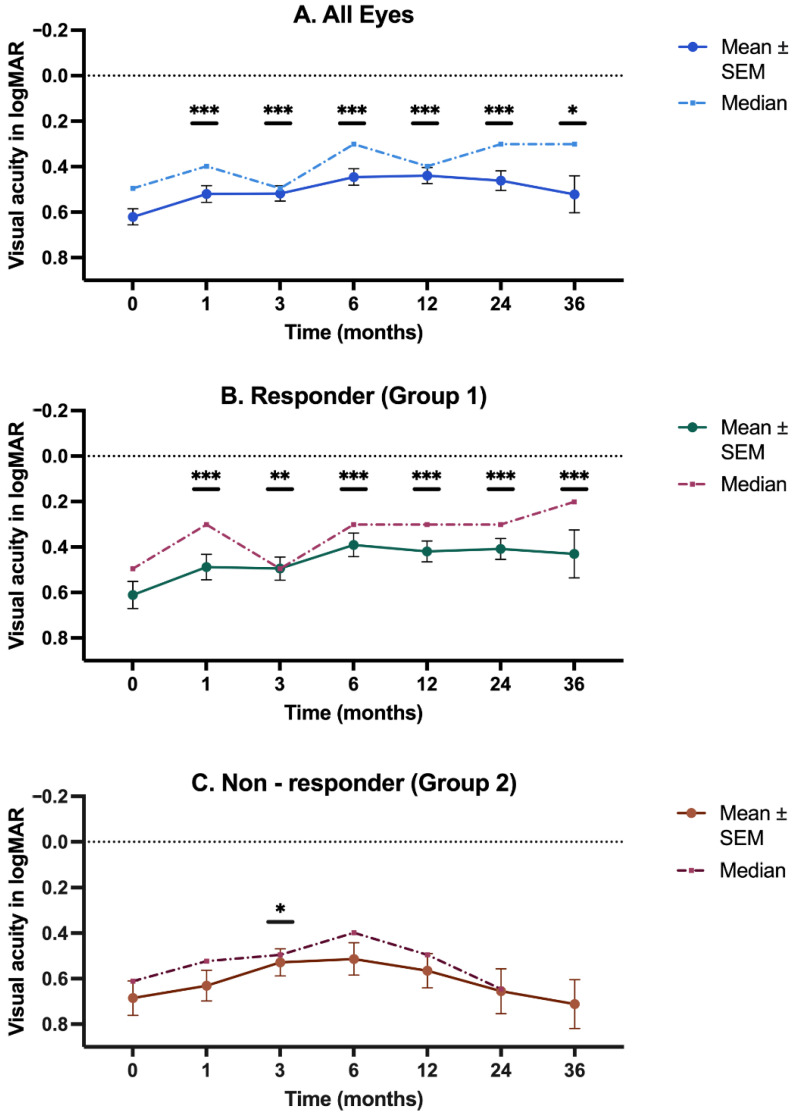
The temporal progression of visual acuity (VA). The figure shows the progression of VA measured at various time points over 36 months. (**A**) The temporal progression of VA in all eyes; (**B**) the temporal progression of VA in the responder group (Group 1); (**C**) the temporal progression of VA in the non-responder group (group 2). SEM = standard error of the mean. * *p* < 0.05, ** *p* < 0.01, and *** *p* < 0.001.

Figure 2A (all eyes) revealed a significant improvement in VA at one and six months compared to baseline (*p* < 0.001), despite a temporary decrease at Month 3. However, VA still remained above the baseline value at Month 3. After Month 6, VA only showed moderate fluctuations.

Group 1 showed a rapid improvement in VA 4 weeks after injection (*p* < 0.001), a decrease in Month 3 (*p* = 0.001), and an increase again in Month 6. Thereafter, the VA remained stable with statistically significant values at all follow-up months (*p* < 0.001). Median values increased from 0.49 logMAR at Month 0 to 0.30 logMAR by Months 6, 12, and 24 and was 0.20 logMAR at Month 36 (Figure 2B).

In Group 2, baseline VA was slightly worse compared to Group 1. There was an initial increase in VA, though it was not as marked as the one seen in Group 1. The increase was only significant at Month 3 (*p* = 0.01). Although it continued to increase up to Month 6, a deterioration followed until Month 36, reaching a value slightly worse than the baseline value (Figure 2C).

### 3.4. Changes in Intraocular Pressure

**Figure 3:**
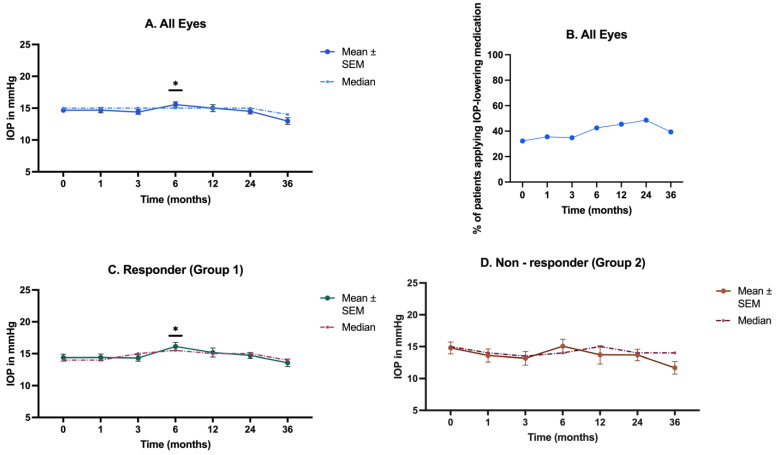
IOP changes and IOP medication usage over time. The figure shows the progression of IOP measured at various time points over 36 months. (**A**) The temporal progression of IOP in all eyes; (**B**) the percentage of patients applying IOP-lowering medication over 24 months in all eyes; (**C**) the temporal progression of IOP in the responder group (Group 1); (**D**) the temporal progression of IOP in the non-responder group (Group 2). IOP = intraocular pressure; SEM = standard error of the mean. * *p* < 0.05.

In terms of intraocular pressure (IOP) (Figure 3A), the median value was 15 mmHg at the initiation of therapy for all eyes. At Month 6, a significant increase in IOP was observed (*p* = 0.028). By Month 12, the average intraocular pressure normalized; however, there was a notable rise in prescribed local IOP therapy, increasing from 32% at baseline to 43% at Month 6. By Month 24, 49% of eyes had been prescribed medication (Figure 3B). An analysis using the GEEs revealed that over time, the probability of a patient taking medication increased significantly (*p* = 0.032).

### 3.5. Morphological Characteristics in Optical Coherence Tomography

The morphological characteristics assessed through OCT imaging refer to the extent and specific location of ME, the extent of damage to the ELM and IS/OS zone, and the presence or absence of gliosis. Table 2 revealed that before therapy, the non-responder group had a significantly higher percentage of eyes with a defect in the ELM and IS/OS zone (*p* < 0.001), with almost 90% of eyes being affected. In contrast, only 42% of eyes from Group 1 were affected. The majority of all eyes had concomitant epiretinal gliosis before treatment (58.5%). The ME was either intraretinal or involved both intraretinal and subretinal regions. Only rarely was the edema was found to be exclusively subretinal.

### 3.6. Pars Plana Vitrectomy vs. Non-Pars Plana Vitrectomy

**Figure 4:**
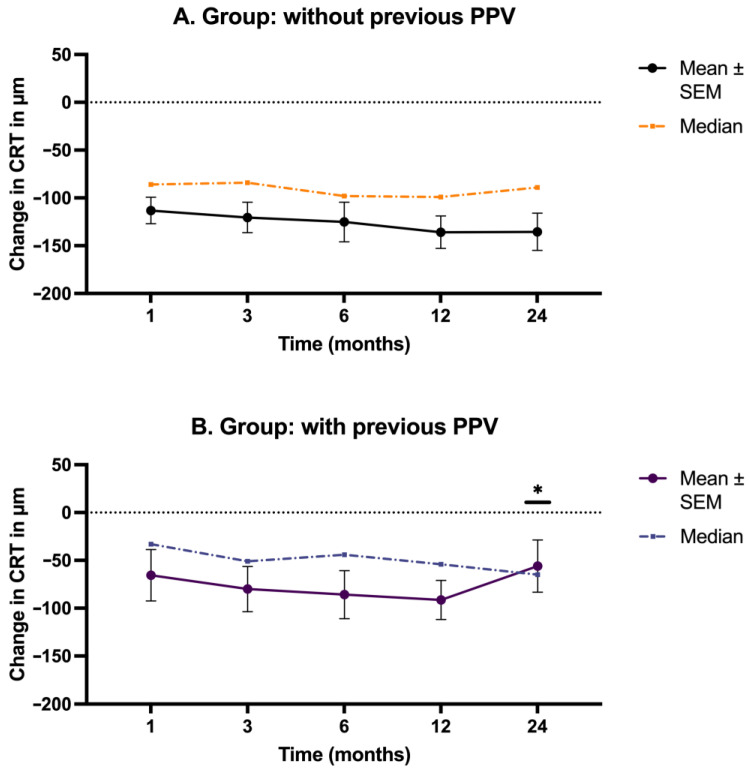
CRT change in non-PPV and PPV eyes. Figure 4 illustrates the magnitude of the reduction (compared to the baseline value at Month 0) in CRT at various time points over 24 months in two different types of patients: (**A**) those without a previous PPV and (**B**) those with previous PPV. CRT = central retinal thickness; PPV = pars plana vitrectomy; SEM = standard error of the mean. * *p* <0.05.

Figure 4 and Table S1 (Supplementary Materials) show that patients with a previous PPV procedure had a milder decrease in CRT than patients without a previous PPV procedure. The difference in the mean CRT decrease between the non-PPV and PPV patients was 47.55 µm (Table S1, Supplementary Materials). This pattern can be observed in the first 12 months of follow-up. In Month 24, there was a statistically significant increase in CRT in patients with previous PPV (*p* = 0.046).

## 4. Discussion

Our study analyzed the real-world effects of FAc implants in patients from a uveitis referral center in Germany, focusing on clinical aspects scarcely addressed in the existing scientific literature. These included differences in the response to therapy in patients with or without a previous PPV, the characteristics of the non-responder group, and a comparison between FAc and DEX therapies. These data are important for understanding the relative efficacy and suitability of these treatments in diverse clinical scenarios. 

### 4.1. Fluocinolone Acetonide vs. Dexamethasone Implants

Compared to the DEX implant, for which peak clinical effects occur between 3 and 6 months, the FAc implant shows prolonged therapeutic efficacy lasting for up to 36 months. This is reflected in the majority of patients and has been determined as a responder group [16,22,35,36]. Subsequently, this longer effect not only lessens the need for frequent re-injections but also alleviates the psychological burden felt by patients in anticipation of an invasive procedure [37]. Complications from invasive procedures are likely reduced with FAc due to less frequent re-injections and the use of smaller 25-gauge needles [38,39]. In contrast, DEX implants require larger 22-gauge needles, which can cause more damage during implantation [40].

Moreover, it has been shown that pharmacokinetics differ between DEX and FAc. The fluocinolone acetonide 0.19 implant releases its active ingredient in a first-order manner, with sustained daily release into the vitreous humor over a 36-month period. Together with a stronger affinity to the corticosteroid receptor compared to DEX, it results in a strong and durable clinical effect [25]. The glucocorticoid signaling pathway has an important role in anti-inflammatory responses. Glucocorticoid receptors are expressed almost exclusively in Müller cells, with the main glial cell type found in the retina [41]. 

Continuous activation of glucocorticoid receptors inherently downregulated its expression through an autoregulatory mechanism, which eventually leads to glucocorticoid resistance. This in turn impairs the anti-inflammatory and anti-edematous response to endogenous ligands or exogenous agonists [41,42,43]. 

The importance of a prolonged and stable effect on clinical outcomes has been repeatedly emphasized. A study conducted by Chakravarthy et al. assessed the influence of macular fluid volume fluctuations on the preservation of VA during anti-VEGF therapy in eyes with recurrent inflammation. It showed that the eyes that experienced the most fluctuations in retinal thickness had significantly worse VA after two years than those with less retinal thickness fluctuation [24].

As shown in our results, FAc does not allow for strong fluctuations in CRT. However, the opposite is true with DEX implants as they have a notably shorter duration of efficacy than FAc, and therefore, a higher fluctuation in macular fluid volume can be anticipated [35], especially during the time in which effects diminish and DEX is re-injected, and it would be expected to occur with repeated DEX injections. 

This calls into question whether permitting considerable ME fluctuation could worsen the outcome measures of the non-responder group who received a significantly higher number of DEX implants. This raises an important clinical consideration as to whether FAc should be chosen as the initial treatment in these patients, either as a monotherapy or in combination with other treatments. Interestingly, no significant difference was found in the percentage of patients receiving systemic therapy when we compared our two groups, which may suggest it has only a limited therapeutical impact in the non-responder group studied.

### 4.2. Real-World Effect of Fluocinolone Acetonide Implant on Central Retinal Thickness, Visual Acuity, and Intraocular Pressure

Our study found statistically significant improvements in CRT and VA over 36 months (*p* < 0.001). By Month 24, the sample size in Group 1 decreased by four patients, which adds reliability and statistical power to these data. The improvement observed in VA as a consequence of the therapy with FAc aligns with findings reported in previous studies [17,44,45].

An increase in IOP was observed at Month 6 (*p* = 0.02). All patients with increased IOP received a topical treatment which was successful in all individuals. It must be emphasized that even before FAc administration, 32% of eyes had already been prescribed IOP-lowering medication. In patients with a known steroid response and a pre-damaged optic nerve, the use of sustained-release intravitreal CSs such as the FAc implant should be reconsidered. The increase in IOP is a well-known adverse effect of the FAc implant and other intravitreal CSs, as reported in other studies [17,44,46]. Nonetheless, the IOP increase is manageable through conservative therapy in almost all cases [17,44]. 

### 4.3. Non-Responder Group (Group 2)

The demographic analysis revealed that the non-responders were, on average, 8 to 9 years older than the responders. This difference suggests that non-responsiveness may result from a longer duration of disease. Extended disease periods can lead to chronic inflammation along with ME fluctuations and potentially irreversible complications [1].

Non-responders also received significantly more prior injections of DEX. This observation raises the question as to whether the patients in the non-responder group developed “resistance” to the therapy with DEX through the saturation of the CS receptors. The effectiveness of FAc therapy seems influenced by disease duration and the patient’s age which, in turn, affects outcome measures and treatment needs.

The responder group showed a drastic decrease in CRT within one month and maintained a stable, low CRT thereafter (between 250 and 300 µm). This was accompanied by a steady improvement in VA. In contrast, the non-responder group only showed a mild initial decrease in CRT and the improvement in VA was delayed, with a significant increase seen in Month 3. 

Furthermore, the long-term decline in VA was greater than the initial improvement. This observation raises a question as to what predictors or biomarkers may be used to indicate a possible limited response to treatment. 

Almost 90% of patients in the non-responder group had a defective ELM and IS/OS zone on OCT. This represents another significant difference from the responder group. It seems that the outcome measures of patients with a defective ELM and IS/OS zone are worse. This finding was also described in patients with diabetic ME [47]. As a consequence of the limited response in this patient group, questions remain regarding how to improve clinical outcomes. Again, the underlying mechanism of intravitreal corticosteroids might be something to consider in future studies. 

Interestingly, DEX and FAc activate distinct gene expression profiles in human trabecular meshwork cell lines. DEX was found to regulate transcripts related to RNA post-transcriptional modifications in TM 86 cells and was involved in histone methylation in TM 93 cells. FAc, however, was found to modulate genes associated with lipid metabolism in TM 86 cells and genes implicated in the cell cycle in TM 93 cells [25]. FAc might therefore be more suitable as an initial therapy in non-responders due to its influence on cellular proliferation and repair mechanisms, possibly leading to improved tissue integrity and reduced pathological changes in these eyes.

### 4.4. Fluocinolone Acetonide Implant Durability and Effect on Patients with Previous Pars Plana Vitrectomy

Concerning the efficacy of the implant, we can add valuable information on vitrectomized patients. A more attenuated decrease in CRT compared to baseline was seen in patients with prior PPV at all follow-up visits. There was a significant difference at Month 24 (*p* = 0.046), with a substantial increase in CRT in patients with previous PPV. This increase demonstrates that the efficacy of therapy was shorter in comparison to patients without previous PPV in which CRT remains at a stable value compared to the previous follow-up month. Considering the substantial number of eyes with prior PPV (38 eyes (28.14%)) in the cohort, the observed effect is well supported. Notably, there was no statistically significant difference between the number of eyes in the responder and non-responder groups regarding previous PPV.

The vitreous gel generally slows down metabolic processes and potentially protects the eye against the unwanted fast release of medication. It can be expected that due to the loss of this reservoir’s structure, medication is released faster in PPV eyes, resulting in shorter effects [48,49,50,51]. This was observed with other medications in multiple studies. For example, two studies showed that the clearance of triamcinolone was accelerated [52,53] in patients who underwent PPV. Reduced half-lives for drugs in vitrectomized eyes were also described for bevacizumab in human eyes and ranibizumab and aflibercept in monkey eyes [54,55]. Studies on DEX effects vary: Çevik et al. observed a statistically significant decrease in foveal thickness for six months in patients without PPV but for only up to three months in those with PPV [48]. Conversely, Medeiros et al. reported statistically significant improvements in BCVA and CRT up to six months in both vitrectomized and non-vitrectomized patients [56]. 

A study that examined the FAc treatment for DME contrasted therapy outcomes between PPV patients and non-PPV patients. It suggested that a possible mechanism of action that led to a reduced effect of therapy in PPV patients was the presence of a pre-retinal hyper-reflective tissue. The study found pre-retinal hyper-reflective tissue more frequently in patients with PPV, and it may act as a barrier that impairs the diffusion of FAc to the retina, causing an accumulation of fluid at the macula [57]. 

The exact mechanism of action of the FAc implant in vitrectomized eyes is not completely clear. We assume, however, that the continuous release of the steroid is in favor of constant exposure to Müller cells as responsive targets. 

### 4.5. Limitations

This study’s retrospective nature led to limitations such as follow-up data not always being complete and potential recall bias, which was minimized by double-checking collected information. Assessing flare-ups was difficult due to incomplete data regarding vitreous haze, which hindered the fluorescein angiography assessment. Since many patients had already undergone cataract surgery, we were limited to evaluating cataract formation as an adverse event. The effect of the type of uveitis on patient outcome measures was not statistically evaluated because of the small numbers of eyes in some categories. In some cases, both affected eyes of the same patient were included in the analysis. While this approach reflects real-world clinical practice, it is important to acknowledge that the systemic status of the patient may influence the course of the disease in both eyes in a similar manner.

## 5. Conclusions

The FAc implant effectively stabilizes CRT, minimizes fluctuations, and enhances VA for up to 36 months. Elevations in IOP were successfully managed. Older age and a defect in the ELM and IS/OS zone may negatively affect therapeutic outcomes. Data also showed that individuals who underwent a PPV experienced a less pronounced reduction in CRT, and the effectiveness of the implants seemed to diminish more quickly. 

## Figures and Tables

**Table 1:** Demographics and clinical characteristics. This table represents the demographics and clinical characteristics of all eyes, responders (Group 1), and non-responders (Group 2). The *p*-value represents the significance of the differences between the two groups. “Other” refers to patients diagnosed with pseudophakic cystoid macular edema (PCME) and uveitis associated with juvenile idiopathic arthritis (JIA).

	All Eyes	Responder (Group 1)	Non-Responder (Group 2)	*p*-Value (Group 1 vs. Group 2)
N (eyes)	135	61	29	
Age (years) Mean ± SD (Median)	63.47 ± 15 (67.61)	60.37 ± 15.48 (65.17)	68.00 ± 16.46 (74.07)	0.0046 *
Sex (female/male)	92/43 (68.14%/31.85%)	41/20 (67.21%/32.78%)	18/11 (62.07%/37.93%)	0.8083 ^
N° of previous DEX implants, Mean ± SD (Median)	5.95 ± 4.52 (5)	5.76 ± 4.34 (5)	7.96 ± 4.96 (7)	0.0487 *
Systemic immunomodulatory therapy (yes/no)	56/79 (41.48%/58.52%)	25/36 (40.98%/59.02%)	14/15 (48.27%/51.17%)	0.6709 ^
PPV (yes/no)	38/96 (28.14%/71.11%)	15/46 (24.59%/75.40%)	8/20 (27.59%/68.96%)	0.8904 ^
Lens state (clear/cataract/pseudophakic)	3/15/117 (2.22%/11.11%/86.67%)	2/2/57 (3.28%/3.28%/93.44%)	1/4/24 (3.45%/13.79%/82.76%)	/ °
Anterior uveitis	14 (10.37%)	8 (13.11%)	5 (17.24%)	/ °
Intermediate uveitis	47 (34.81%)	21 (34.43%)	11 (37.93%)	
Posterior uveitis	46 (34.07%)	18 (29.51%)	7 (24.14%)	
Panuveitis	12 (8.89%)	5 (8.20%)	3 (10.34%)	
Other	16 (11.85%)	9 (14.75%)	3 (10.34%)	

DEX = dexamethasone; PPV = pars plana vitrectomy; SD = standard deviation. * Wilcoxon signed-rank test; ^ Fisher‘s Exact test; / ° No Chi-squared test possible—sample sizes too small in individual categories; mean  ±  SD (median); number of eyes = N (%).

**Table 2:** Morphological characteristics. The table represents the morphological characteristics observed on OCT images from the OCT Heidelberg electronic medical database. The table displays data on the presence and type of ME, the integrity of the ELM + IS/OS Zone, and the occurrence of gliosis across 3 different groups: all eyes, responders (Group 1), and non-responders (Group 2). “Missing data” refers to the fact that the OCT was not readable because of reduced quality, e.g., due to extensive inflammation.

**1. OCT Macular Edema**				
**Condition**	**All Eyes ** **N (%)**	**Responder (Group 1) ** **N (%)**	**Non-Responder (Group 2)** **N (%)**	***p*** **-Value** **(Group 1 vs. Group 2)**
None (0)	7 (5.2%)	4 (6.7%)	0 (0%)	/ °
Intraretinal (1)	63 (46.7%)	29 (48.3%)	11 (39.3%)	
Subretinal (2)	1 (0.7%)	0 (0%)	0 (0%)	
Both (3)	61 (45.2%)	27 (45%)	17 (60.7%)	
Missing Data	3 (2.2%)	1 (1.7%)	1 (3.6%)	
Total	135	60	28	
**2. OCT ELM and IS/OS Zone**				
**Condition**	**All Eyes** **N (%)**	**Responder (Group 1)** **N (%)**	**Non-Responder (Group 2)** **N (%)**	***p*** **-Value** **(Group 1 vs. Group 2)**
Intact (0)	55 (40.7%)	34 (57.6%)	3 (10.7%)	0.000025 ^
Defect (1)	76 (57.6%)	25 (42.4%)	25 (89.3%)	
Missing Data	4 (3.0%)	2 (3.4%)	1 (3.6%)	
Total	135	59	28	
**3. OCT Gliosis**				
**Condition**	**All Eyes** **N (%)**	**Responder (Group 1)** **N (%)**	**Non-Responder (Group 2)** **N (%)**	***p*** **-Value** **(Group 1 vs. Group 2)**
No (0)	55 (40.7%)	25 (41.0%)	7 (25.0%)	0.162188 ^
Yes (1)	79 (58.5%)	36 (59.0%)	21 (75.0%)	
Missing Data	1 (0.7%)	0 (0.0%)	1 (3.6%)	
Total	135	61	28	

OCT = optical coherence tomography; ELM = external limiting membrane; IS = inner segment; OS = outer segment. ^ Fisher’s Exact test. / ° No Chi-squared test possible—sample sizes too small in individual categories.

## Data Availability

Data are available upon reasonable request.

## Supplementary Materials

The following supporting information can be downloaded at https://www.mdpi.com/article/10.3390/biomedicines12051106/s1, Table S1. Linear Regression Analysis results: PPV vs. no previous PPV.

## Abbreviations and Acronyms

Anti-vascular endothelial growth factor (anti-VEGF), best corrected visual acuity (BCVA), central retinal thickness (CRT), corticosteroid (CS), dexamethasone (DEX), diabetic macular edema (DME), external limiting membrane (ELM), fluocinolone acetonide (FAc), inner segment/outer segment (IS/OS), intraocular lens (IOL), intraocular pressure (IOP), macular edema (ME), neovascular age-related macular degeneration (nAMD), non-infectious uveitis (NIU), non-responder group (Group 2), optical coherence tomography (OCT), pars plana vitrectomy (PPV), pseudophakic cystoid macular edema (PCME), responder group (Group 1), standard deviation (SD), standard error of the mean (SEM), tumor necrosis factor-alpha (TNF-α), visual acuity (VA), inner limiting membrane (ILM), retinal pigment epithelium (RPE), and juvenile idiopathic arthritis (JIA).
