# Supplementary material for: Fluocinolone Acetonide Implant for Uveitis: Dissecting Responder and Non-Responder Outcomes at a Tertiary Center
Source: Biomedicines. 2024 May 16;12(5):1106. doi: 10.3390/biomedicines12051106 (PMC11117563; doi:10.3390/biomedicines12051106)
Supplement: Supplementary file 1 [file biomedicines-12-01106-s001.zip › biomedicines-2973589-supplementary.pdf]

Table S1. Linear Regression Analysis Results PPV vs No Previous PPV.

In the linear regression analysis, the intercept (β0) represents the mean CRT change for the non-PPV group. The PPV coefficient (β1) indicates the difference in mean CRT change between the PPV and non-PPV groups.

CRT = central retinal thickness, PPV = pars plana vitrectomy

Month	Intercept (β0)	PPV Coefficient (β1)	*p*-Value
1	−113.13	47.55	0.0999
3	−120.43	40.49	0.1685
6	−125.20	39.38	0.2754
12	−135.88	44.47	0.1346
24	−135.45	79.45	0.0466
